# A Case of Concomitant Perforated Acute Cholecystitis and Pancreatitis

**DOI:** 10.1155/2013/263046

**Published:** 2013-07-16

**Authors:** Marlon Perera, Toan Pham, Sumeet Toshniwal, Yasmin Lennie, Steven Chan, Nezor Houli

**Affiliations:** Department of Surgery, Western Health, Footscray, Melbourne, VIC 3012, Australia

## Abstract

*Introduction*. Concomitant cholecystitis and gallstone pancreatitis is an infrequent clinical encounter, reported sparsely in the literature. Concurrent acute cholecystitis and pancreatitis complicated by gall bladder perforation has not been reported before. *Presentation of Case*. We report a 39-year-old female presenting with concomitant cholecystitis and acute pancreatitis, complicated by gallbladder perforation. *Discussion*. There is much controversy surrounding the timing of cholecystectomy following gallstone pancreatitis, with the recent literature suggesting that “early” operation is safe. In the current case, gallbladder perforation altered the “routine” management of gallstone pancreatitis and posed as a management dilemma. *Conclusion*. Clinical judgement dictated timing of operative management and ultimately cholecystectomy was performed safely.

## 1. Case Report

A 39-year-old female, previously well, was admitted with a diagnosis of acute pancreatitis with concurrent acute calculus cholecystitis. The patient presented with a one-day history of acute onset sharp pain in the epigastrium, associated with nausea and vomiting. She was exquisitely tender in the epigastrium and right upper quadrant, with no peritoneal signs on examination. Laboratory investigations showed a white cell count of 14.2 × 10^6^/L, with 81% neutrophils, 28 U/L bilirubin, 237 U/L ALT, 192 U/L AST, 145 U/L ALP 171 U/L GGT, lipase 1445 U/L, and LDH 630 U/L, and serum glucose was 7.9 mmoL/L. The patient was classed as grade 1 pancreatitis using Ranson's criteria [1]. Biliary ultrasound on admission showed acute cholecystitis, with gallbladder wall thickness of 5 mm and multiple calculi, the largest at 6 mm and no evidence of biliary tree dilation.

**Figure 1:**
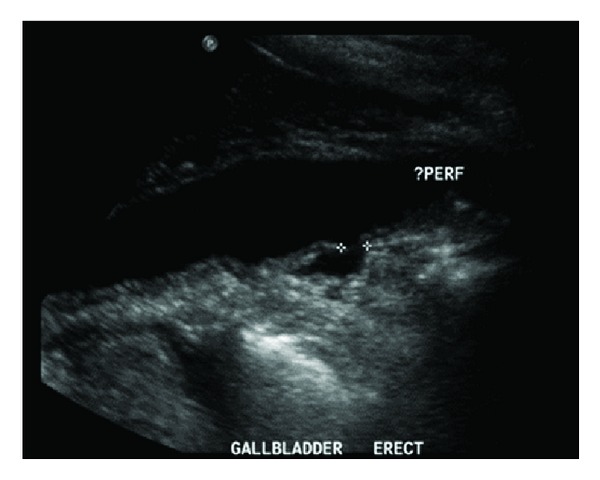
Ultrasonographic evidence from the repeat ultrasound of gallbladder perforation.

**Figure 2:**
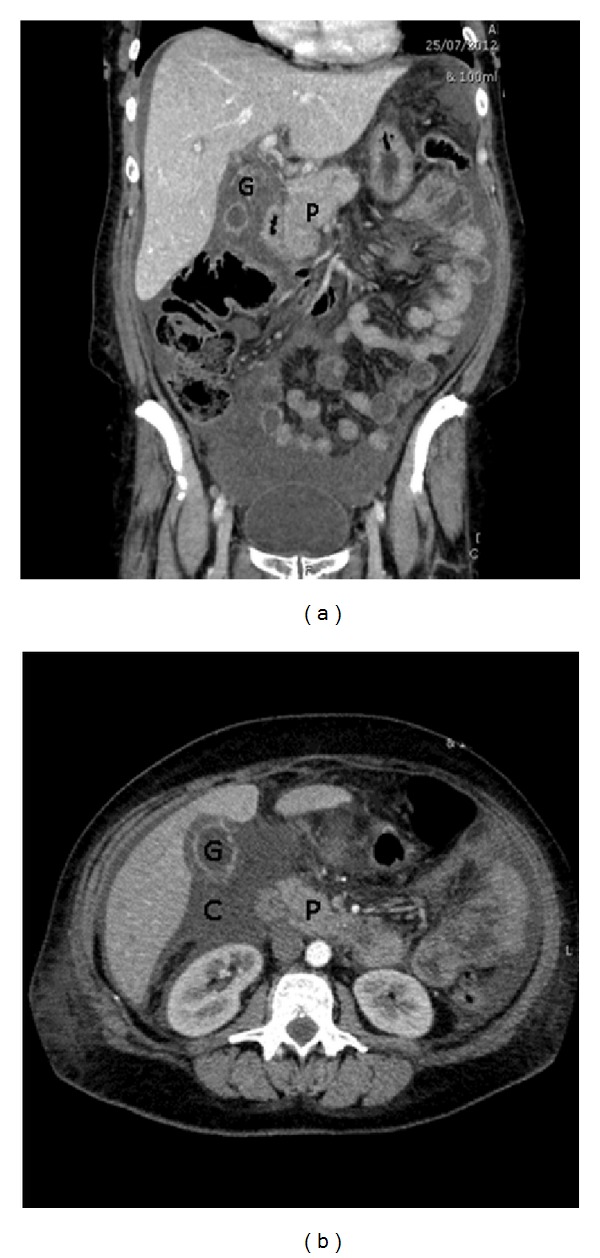
CT of the abdomen illustrating peripancreatic fat stranding and free fluid around the gallbladder. G: Gallbladder, P: Pancreas, C: pericholecystic collection.

The patient was initially managed with empiric parental antibiotics; ceftriaxone 1 g daily and metronidazole 500 mg 8 hourly. On day 2 of admission, liver function became increasingly deranged, with bilirubin increasing to 77 U/L. The patient complained of acute exacerbation of pain and repeat sonography showed worsening cholecystitis with a high suspicion of a small gallbladder posterior wall perforation, associated with an increase of pericholecystic fluid collection (Figure 1). Computerized tomography (CT) of the abdomen and Pelvis showed large amounts of free fluid within the gallbladder fossa, confirming gallbladder perforation. There was marked fat stranding in the upper abdomen surrounding the pancreas and extensive free fluid in the perihepatic region and perisplenic region, upper abdomen, iliac fossae and Pouch of Douglas (Figure 2). 

**Figure 3:**
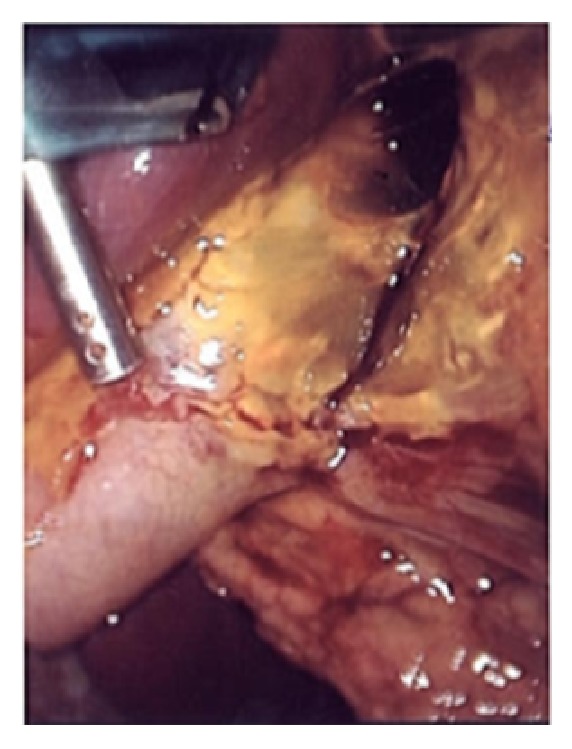
Intra-operative confirmation of posterior wall gallbladder perforation.

The patient underwent a semielective laparoscopic cholecystectomy. Intraoperatively, the diagnosis of perforated necrotic cholecystitis was confirmed with 600 mls of bilious free fluid within the peritoneum (Figure 3). Intra-operative cholangiogram was otherwise unremarkable, though a prominent pancreatic duct was noted. There was no obvious common bile duct obstruction. The patient improved clinically over the postoperative period with rapid improvement in inflammatory markers, enabling the patient to be discharged three days postoperatively.

## 2. Discussion

Concomitant acute cholecystitis and gallstone pancreatitis is common clinical constellation that has been documented in the literature. However, to the authors' knowledge, no such case of gallbladder perforation in conjunction with pancreatitis has been previously documented. 

The current case had clear biochemical and radiological confirmation of both pancreatitis and cholecystitis. Pancreatitis was established on presentation by clinical symptomatology and serum lipase exceeding four times the upper limit. Later, radiological evidence of peripancreatic fat stranding on CT and dilated pancreatic duct on intraoperative cholangiogram [2] confirmed the presence of pancreatitis.

Calculus cholecystitis with gallstone pancreatitis has been well documented. Danielle Dietz [3] reported that up to 40% of patients undergoing interval cholecystectomy for gallstone pancreatitis have some histological evidence of acute cholecystitis. A proposed causation is that gall bladder inflammation is secondary to pancreatic reflux in the context of preceding pancreatitis. Further, Sanchez-Ubeda et al. [4] reported 29 cases of gallstone pancreatitis associated with chronic cholecystitis, 24 of which showed evidence of acute inflammatory changes. Sanchez-Ubeda et al. reported that the majority of the cases of pancreatitis were classed as mild, represented by an isolated increase in amylase or mild pancreatic oedema.

Current practice for uncomplicated mild biliary pancreatitis is controversial. Historically, early cholecystectomy has been avoided due to the belief that the procedure would be complicated by difficult dissection caused by oedema. However, recent literature suggests early cholecystectomy can be performed with minimal change in morbidity and mortality [5]. Unfortunately there is no consensus on the definition of “early” cholecystectomy. Currently, the International Association of Pancreatology suggests that cholecystectomy at the time of clinical and biochemical recovery of pancreatitis [6]. Conversely, the American Gastroenterology Association [7] and the British Society of Gastroenterology [8] recommend surgical intervention within 2 to 4 weeks postdischarge. 

Patients managed by interval cholecystectomy are at significantly increased risk of representation due to biliary disease, whether biliary colic or repeat pancreatitis. Ito et al. reviewed patients who underwent interval cholecystectomy and noted that up to 31% of patients had recurrent gall-stone events within 2 weeks of discharge [9]. These findings have been reproducible, as reported in a systematic review recently [10]. Other advocates for early cholecystectomy including Aboulian et al. [5] demonstrated that cholecystectomy can safely be performed within 48 hours of admission in the context of pancreatitis—regardless of the resolution of symptoms of biochemical abnormalities. Aboulian et al. reported a significantly decreased hospital stay with no difference in rates of conversion or surgical complications. 

In the current case, radiological confirmation of perforated cholecystitis in the setting of acute pancreatitis was made. These findings ultimately dictated the timing of early cholecystectomy. 

This is a rare case report of concomitant perforated cholecystitis and acute pancreatitis. We conclude that in the emergency setting, laparoscopic cholecystectomy may be performed in the context of pancreatitis.
